# Uniform Temperature Dependency in the Phenology of a Keystone Herbivore in Lakes of the Northern Hemisphere

**DOI:** 10.1371/journal.pone.0045497

**Published:** 2012-10-05

**Authors:** Dietmar Straile, Rita Adrian, Daniel E. Schindler

**Affiliations:** 1 Limnological Institute, University of Konstanz, Konstanz, Germany; 2 Leibniz-Institute of Freshwater Ecology and Inland Fisheries, Berlin, Germany; 3 School of Aquatic and Fishery Sciences, University of Washington, Seattle, Washington, United States of America; Macquarie University, Australia

## Abstract

Spring phenologies are advancing in many ecosystems associated with climate warming causing unpredictable changes in ecosystem functioning. Here we establish a phenological model for *Daphnia*, an aquatic keystone herbivore based on decadal data on water temperatures and the timing of *Daphnia* population maxima from Lake Constance, a large European lake. We tested this model with long-term time-series data from two lakes (Müggelsee, Germany; Lake Washington, USA), and with observations from a diverse set of 49 lakes/sites distributed widely across the Northern Hemisphere (NH). The model successfully captured the observed temporal variation of *Daphnia* phenology in the two case study sites (r^2^ = 0.25 and 0.39 for Müggelsee and Lake Washington, respectively) and large-scale spatial variation in the NH (R^2^ = 0.57). These results suggest that *Daphnia* phenology follows a uniform temperature dependency in NH lakes. Our approach – based on temperature phenologies – has large potential to study and predict phenologies of animal and plant populations across large latitudinal gradients in other ecosystems.

## Introduction

One of the most evident effects of climatic change during recent decades is the advancement of spring phenological events in terrestrial [1], [2], marine [3], [4], and freshwater ecosystems [5]–[7]. Phenological changes can affect ecosystem functioning as they can disrupt food web interactions [8], [9]. In freshwater ecosystems, cladocerans of the genus *Daphnia* are keystone herbivores that are important drivers of algal seasonal succession and community composition [10]. As important prey themselves, daphnids are crucial for survival and growth of small fish. In evolutionary terms fish reproduction is often timed to allow juvenile fish to exploit the seasonal *Daphnia* maximum. Consequently, *Daphnia* phenology has important implications for ecological and evolutionary dynamics in lake ecosystems.

The timing of the *Daphnia* maximum has been shown to be strongly associated with large-scale atmospheric oscillations, e.g. the North Atlantic Oscillation in Europe [5], [11], [12] and the Pacific Decadal Oscillation in Northern America [7]. Furthermore, *Daphnia* peak timing is strongly correlated with latitude in Northern America and has been suggested to occur when surface water temperatures reach 18.5°C [13]. This suggests a strong control of *Daphnia* population dynamics by water temperatures making their phenology sensitive to climate warming.

Mechanistic simulation models of various complexity have been developed to examine *Daphnia* phenology [11], [14], [15]. These models emphasize the importance of water temperature but also address the potential importance of food phenology, mortality rates due to predators, and *Daphnia* life cycle strategies [11], [14], [15]. These mechanistic models allow prediction of *Daphnia* seasonal population dynamics under a variety of environmental conditions. However, those conditions and their possible changes with ongoing global warming need to be specified in these models. Hence, there is need for empirical models to allow for general predictions of the effects of changing thermal regimes on *Daphnia* phenology.

**Figure 1:**
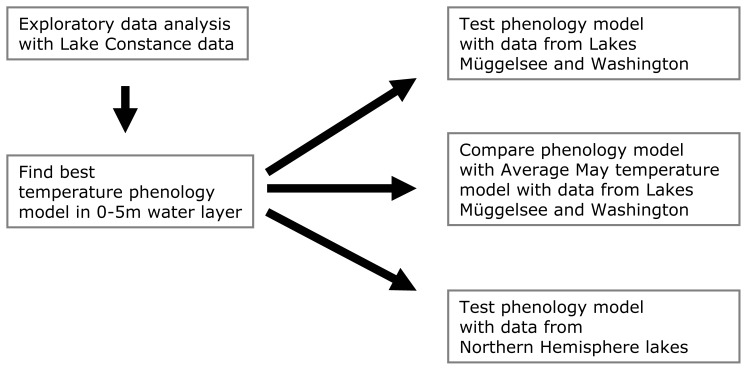
Flow chart of statistical analyses performed in this study.

In previous studies, *Daphnia* spring dynamics have been related to water temperatures averaged across specific times of the year (e.g., [6], [7], [16]). However, this approach requires that these time periods need to be determined specifically for each lake owing to differences in vernal warming among lakes due to their depth, elevational and latitudinal setting. This suggests that an approach based on temperature averages, although very useful for specific studies, does not allow predictions across lakes generally, e.g. those with different morphometries or at different latitudes. An alternative approach is to use temperature phenology as a predictor for *Daphnia* seasonal dynamics. Here we develop and test such a model with data from lakes across the Northern Hemisphere (see Figure 1 for a flow chart of analyses performed). We first develop a linear model to describe the temperature dependency of the timing of *Daphnia* peak seasonal densities in Lake Constance, a large and intensively studied perialpine lake in central Europe (thereafter LC *Daphnia* phenology model). We then test the LC phenology model with time series data from two other well studied lakes in temperate regions: large, deep Lake Washington, USA and small, shallow Müggelsee, Germany and compare the temperature phenology models with models using average May temperatures as predictors. Finally, we test the LC phenology model with data from a literature survey of *Daphnia* dynamics from 49 lakes/sites across the Northern Hemisphere thereby testing its applicability beyond the climatic range for which it was established.

## Materials and Methods

**Figure 2:**
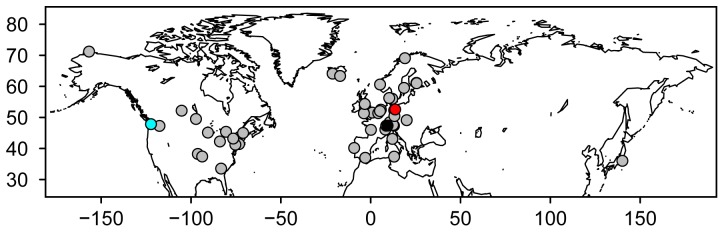
Geographical location of lakes considered in this study. Location of Lake Constance is shown as a black dot. Locations of the two lakes for which temporal variability in *Daphnia* phenology was analysed shown as blue (Lake Washington, US), respectively, red dot (Müggelsee, Germany). Locations of lakes considered in the Northern hemisphere study are shown as grey dots.

*Daphnia* in deep warm-monomitic Lake Constance were sampled weekly (biweekly in 2002) during the spring period from 1979 to 2007 with the exception of 1983 when no data were collected. Samples were collected with a Clarke-Bumpus sampler by vertical hauls from a depth of 140 m in the Überlinger See, a deep and fjordlike appendix of the lake [16]. Water temperature at the sampling station was recorded either with temperature probes at a weekly resolution or by quasi-continuous measurements (every 20 min) from thermistor chains employed at the sampling station [17]. Prior to analysis water temperature data were aggregated and interpolated to provide a daily temporal resolution and a depth resolution of 1 m in the upper 20 m of the water column. Information on the measurements of *Daphnia* abundances and water temperatures in Müggelsee (1980–2007) and Lake Washington (1977–2007) can be found in [6], [7]. No specific permits were required for the described field studies. From a literature survey 66 seasonal dynamics of *Daphnia* and water temperature were derived from 49 different sites (lakes, ponds, and reservoirs) in the Northern Hemisphere ranging from 33° 52′ N to 71° 20′ N latitude and from −1 to 3040 m elevation (Figure 2, see Table S1 for information on the study sites). We chose only sites with a sampling resolution of ≥2 samples per month during the spring/early summer period. To obtain data from these literature studies, relevant figures were scanned at high resolution and digitized. We did not distinguish between the different *Daphnia* species (*D. ambigua, D. cucullata, D. galeata, D. galeata mendotae, D. hyalina, D. longispina, D. parvula* and *D. rosea*) and calculated the total number of *Daphnia* per sampling date and analysed the phenology of these time-series. We also included studies reporting the dynamics of *Daphnia* biomass as *Daphnia* abundance is usually very tightly related to *Daphnia* biomass (e.g. for Lake Constance: r^2^>0.9). For all *Daphnia* seasonal dynamics, the timing of the *Daphnia* late spring/early summer maximum (TD_max_) was defined as the date of maximum abundance/biomass during the period from 1 March until 31 July. For high elevation (>1000 m asl) lakes and/or high latitude lakes (>60°N) we extended the period for maximum abundance/biomass to 30 September.

Differences in a) average TD_max_ and b) the surface temperature at TD_max_ between Lakes Constance, Washington and Müggelsee were analysed by comparing the respective linear models (with lake as a factor) and null-models, both with autocorrelated errors, using a Likelihood Ratio test.

The relationship between *Daphnia* phenology and water temperature phenologies in the Lake Constance time series was analysed in an exploratory study. For all study years we recorded the first day in the year when specific temperatures (from 6 to 20°C with 0.5°C steps) were obtained at the surface and on average within the upper 5 m, upper 10 m and upper 20 m of the water column. As data were normally distributed Pearson correlation analysis was used to relate these timings of water temperatures (TT) to TD_max_ to identify depth layer –temperature combinations with the highest predictive power for TD_max_. For those correlations between TD_max_ and the upper 5 m average water TT which yielded the highest Pearson r, we additionally implemented linear models with autocorrelated errors and compared their performance using AIC corrected for small sampling sizes (AICc) [18].We chose Lake Constance for establishing our baseline model as we expect in this large and deep lake to have the largest variation in temperature-driven change in phenology which should facilitate establishing a generalizable model. Furthermore, spring *Daphnia* dynamics in Lake Constance have been shown to be strongly influenced by water temperatures [16], and there is no evidence that spring *Daphnia* dynamics have been influenced by a mismatch with their algal food source as has been reported for Lake Washington [9]. We also prefer to use Lake Constance as a baseline model over the NH data set as there is no reason to assume that the observed temperature TD_max_ relationship in Lake Constance may be confounded by correlated systematic variation. In contrast, within the Northern Hemisphere data set, TD_max_ variability is expressed mostly along latitude, which is associated with gradients in e.g., temperature, season length, *Daphnia* size [13], and the strength of biotic interactions [19].

The best linear model based on AICc between TD_max_ and the upper 5 m average water TT in Lake Constance was used to predict TD_max_ in Lakes Washington, Müggelsee and in the Northern Hemisphere Lakes data set. As measurements of water temperatures also include errors, we used standardized major axis (SMA) regression to test whether the intercept and slope of the relationship between observed and predicted TD_max_ in these lakes differed from zero and one, respectively [20], to test for systematic bias in predicted TD_max_. We also report the explained R^2^ of the relationships between predicted and observed TD_max_ for these regressions.

In a second approach we used linear models with autocorrelated errors (AR1) a) to determine whether the response to TT differed between Lakes Constance, Washington and Müggelsee, b) to compare the TT models with models predicting *Daphnia* phenology with average May temperatures, and c) to compare the performance of models differing in the independent factors (TT, elevation and latitude), i.e., to test whether TT’s were able to explain a similar part of the variability in TD_max_ as the latitudinal and elevational setting of the lakes in the NH data set. We compare models based on AICc and a measure of Pseudo-R^2^, i.e., the R^2^ between observed values and model predictions. Autocorrelation in our data sets was small and models with autocorrelated errors did only in some cases perform slightly better (based on AICc and residual diagnostics) than the corresponding models without autocorrelated errors. However, for consistency we performed all linear models with considering autocorrelated errors. Statistical models were implemented using the R packages SMATR [21] and NLME [22], the latter with parameter estimation based on maximum likelihood. We used SAS (version 8) [23] for data processing and R (version 2.13.0) [24] for statistical analysis and graphs.

## Results

**Figure 3:**
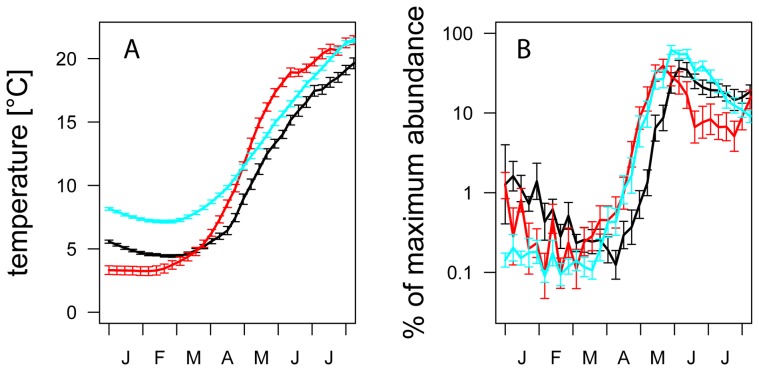
Average seasonalities of A) upper water layer (0–5 m in Lake Constance and Washington, 0 m in Müggelsee) temperatures and B) *Daphnia* dynamics in Lakes Constance (black), Washington (blue), and Müggelsee (red). Prior to averaging data were binned into 7-day periods. To account for differences in absolute abundance and achieve comparability of *Daphnia* dynamics between lakes, *Daphnia* dynamics in each lake and each study year were scaled relative to its maximum abundance during the first 210 days of the year. Scaled data were log-transformed and averaged across study years.

The average development of upper water layer temperatures in the first half of the year differed strongly between the three lakes with long-term data (Figure 3A). January and February temperatures were highest at Lake Washington and lowest at Müggelsee. During April and May vernal warming was fastest for Müggelsee and slowest for Lake Washington. The differences in late winter temperature combined with the differences in vernal warming resulted in highest June water temperatures in Müggelsee, followed by Lakes Washington and Constance (Figure 3A). Average *Daphnia* dynamics were characterized by low and declining abundances during the first three months of the year and started to increase in all lakes around March – April (Figure 3B). During April and May *Daphnia* population increases in all three lakes were remarkably similar and maxima of average population trajectories were reached in early June in Lake Constance and Washington and approximately two weeks earlier in Müggelsee.

The difference in the peak timing of *Daphnia* between Müggelsee and the other two lakes is confirmed when computing the average TD_max_ (Likelihood Ratio Test, L = 13.05, p = 0.0015) (Table 1). However, the surface water temperature at TD_max_ did not differ between lakes (Likelihood ratio Test, L = 4.1, ns) (Table 1). In the northern Hemisphere dataset, TD_max_ varied strongly and ranged from February/March in Florida and Portuguese lakes to August/September in high latitude, and high elevation lakes; the average temperature at TD_max_ was 16.6±4.6°C (mean, std dev).

**Table 1:** Mean timing of the *Daphnia* maximum (TD_max_) and mean surface temperature at TD_max_ in Lakes Constance, Washington and Müggelsee.

	Timing of *Daphnia* maximum (TD_max_)(days since 1 Jan)	Surface Temperature at TD_max_ (°C)
Lake Constance	159±18 (SD)	15.5 (2.5)
Lake Washington	158±18 (SD)	16.7 (2.1)
Müggelsee	140±7 (SD)	16.2 (2.1)

**Figure 4:**
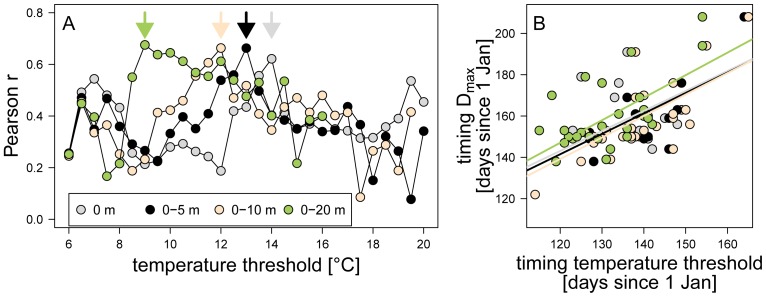
Exploratory analysis of the *Daphnia* phenology - water temperature phenology relationship in Lake Constance. A) Correlation coefficients between the timing of the *Daphnia* maxima and the timing when different water temperatures where first reached in various water column depths in Lake Constance during 1979–2007. Arrows indicate the temperatures with largest Pearson r for each water column depth. B) Relationships between the timing of the *Daphnia* maximum and the timing of those water temperatures with highest Pearson correlation coefficients at the respective depths (see arrows in Fig. 4A). Linear model equations for the TD_max_ and TT’s of 14°C at the surface (14°C_0m_): y = 27.0+0.97*x (Pseudo R^2^ = 0.39 ), 13°C_0–5m_: y = 22.2+0.99*x (Pseudo R^2^ = 0.44), 12°C_0–10m_: y = 14.5+1.04*x (Pseudo R^2^ = 0.44), 9°C_0–20m_: y = 16.2+1.09*x (Pseudo R^2^ = 0.46).

The timing of the *Daphnia* maximum showed a strong relationship to the seasonal timing of water temperature at different depths in Lake Constance (Figure 4). Correlation coefficients of similar magnitudes with different TT’s and water depths are expected due to temporal and spatial autocorrelation of water temperatures. Nevertheless, correlation analyses identified TT-depth combinations with the highest predictive power for TD_max_ (Figure 4A). The highest Pearson correlation coefficients were obtained when relating the TD_max_ to TT of 14°C at the surface, 13°C at 0–5 m, 12°C at 0–10 m, and 9°C at 0–20 m, i.e., with increasing thickness of the surface layer the TT’s at which the highest correlation coefficients with TD_max_ were observed, decreased. This shift in highest correlation coefficients with increasing TT corresponds to decreasing water temperatures with layer thickness. That is, highest correlation coefficients were obtained when surface temperatures reach 14°C and this timing corresponds closely to the timing of 13°C - temperature at 0–5 m depth, of 12°C at 0–10 m depth, and of 9°C at 0–20 m depth (Figure 4B). As our aim was to establish one model useful for predicting TD_max_ both in shallow (i.e. <10 m depth) and deep lakes, we focused on the best linear model for the 0–5 m depth layer, i.e. TT_13, 0–5_:
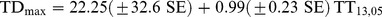
(1)


Note, that based on AICc this model also outperformed (all ΔAIC >4, Table 2) models based on the 12, 12.5 and 13.5 TT’s within the 0–5 m depth layer, i.e. the TT’s which Pearson correlation coefficients ranking next to the 13°C TT (Figure 4A). However, as the 0–5 m layer temperatures did not reach 13°C in some high elevation and high latitude lakes (6 out of 66 lake-years), the prediction of TD_max_ was based on equ. 1 but using the timing of the maximum water temperature achieved in those lakes as the independent variable. In Lake Constance the model with TT_13, 0–5_ explained 44% of the variability in TD_max_ (n = 28, p<0.001, Figure 4B).

**Table 2:** Results of linear models analysing the effects of selected temperatures thresholds (TT) within the 0–5 m depth layer on the timing of TD_max_ in Lake Constance.

Model	TT	intercept	Slope	AICc	Pseudo-R^2^
1	13	22.2(±32.6)	0.99 (±0.23)	1.6	0.44
2	12.5	58.2 (±28.9)	0.75 (±0.21)	5.8	0.36
3	12	71.4 (±30.0)	0.66 (±0.22)	8.8	0.30
4	13.5	73.9 (±32.9)	0.60 (±0.23)	9.1	0.24

All models were calculated with autocorrelated errors.

### Tests with Long-term Data from Müggelsee and Lake Washington

**Figure 5:**
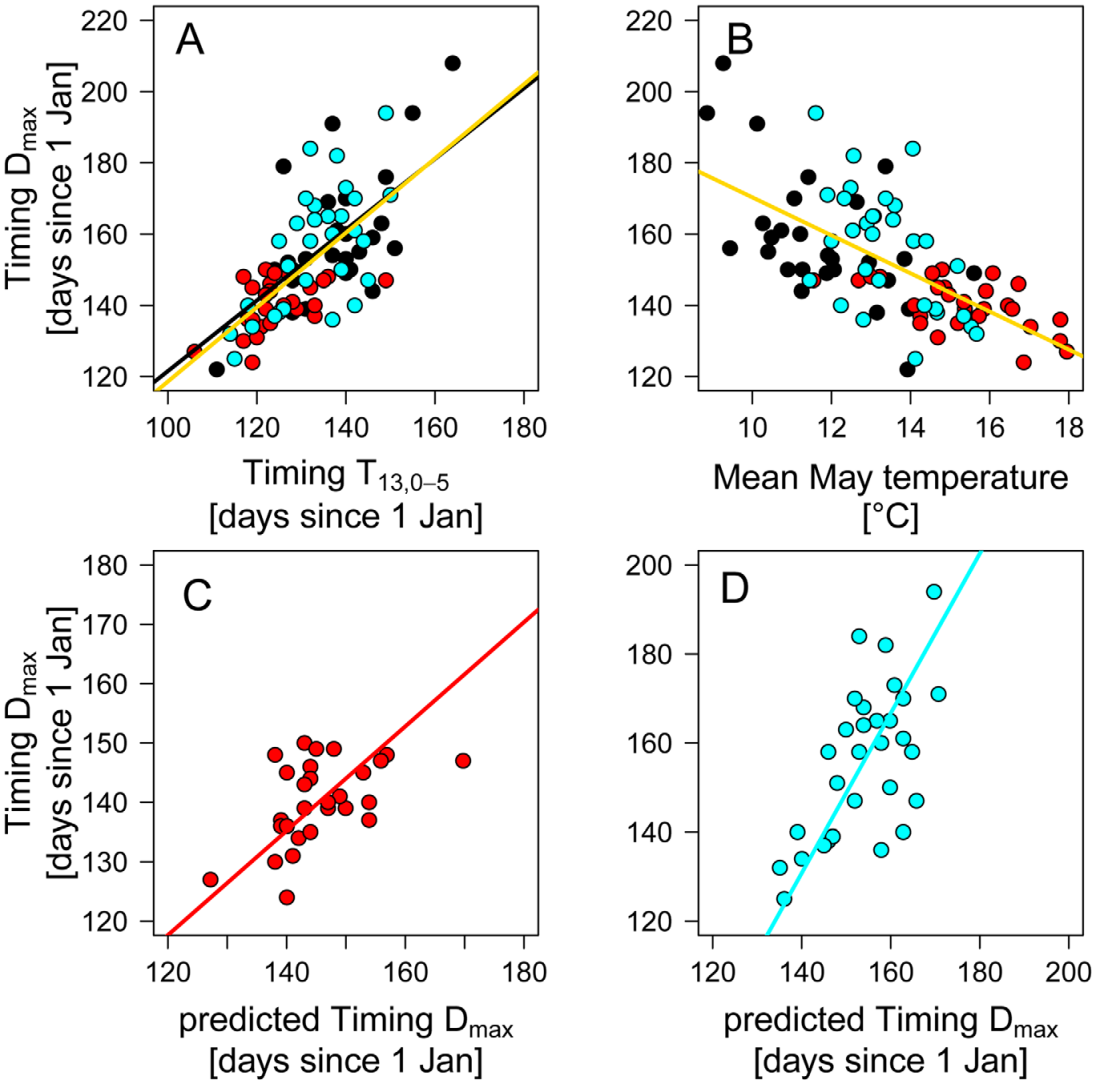
Relationship between the timing of the *Daphnia* maximum and A) the timing when water temperatures reached 13°C, and B) mean May temperatures in the upper 5 m of the water column in Lakes Constance (black dots), Müggelsee (red dots), and Washington (blue dots). Relationship between observed and predicted timing of the *Daphnia* maximum in C) Müggelsee, and D) Lake Washington. The black line in subplot A shows the Lake Constance *Daphnia* phenology model, the yellow line in subplots A and B represent the fits of model 1 and model 4, respectively (Table 2). In subplots C and D lines represent the fits of SMA regression models.

When using data from all three lakes with long-term records (i.e., Müggelsee and Lakes Constance and Washington) TT_13, 0–5_ explained 49% of the variability of TD_max_. Furthermore, the slope and intercept of the relationship (Figure 5A and Table 3) were very close to the relationship observed for Lake Constance data only (equ. 1). Models allowing for lake-specific intercepts and lake-specific intercepts and slopes did not perform better (differences in AICc ≤1, Table 3) than the model without lake-specific parameters. Pseudo-R^2^ differed only slightly between the models (Table 3). Also there was no strong evidence for lake-specific intercepts (F_2,81_ = 1.9, p = 0.15) nor lake-specific-slopes (F_2,81_ = 2.67, p = 0.07).

**Table 3:** Results of linear models analysing the effects of TT_13,0–5_ and average May temperatures (T_May_) on the timing of TD_max_ in Lakes Constance, Müggelsee and Washington.

Model	Factors	AICc	Pseudo-R^2^
1	TT_13, 0–5_	683.4	0.49
2	TT_13, 0–5_, lake	683.7	0.52
3	TT_13, 0–5_, lake, TT_13, 0–5_ * lake	682.7	0.55
4	T_May_	696.3	0.42
5	T_May_, lake	697.5	0.45
6	T_May_, lake, T_May_ * lake	695.5	0.48

All models were calculated with autocorrelated errors.

Similar to TT_13, 0–5_, mean May temperatures were significantly related to TD_max_ (Figure 5B and Table 3) and lake-specific intercepts and slopes did not increase significantly model performance (Table 3). However, models with TT_13, 0–5_ clearly outperformed models with average May temperatures as predictors (Table 3), although Pseudo-R^2^ values of the former were not strongly improved.

As expected from these similar responses of TD_max_ to TT_13, 0.5_, a significant fraction of the interannual variation in TD_max_ of Müggelsee and Lake Washington, could be explained with the TD_max_−TT_13, 0–5_ relationship established for Lake Constance (Figure 5C,D). In Müggelsee this relationship explained 25% of the observed variation in TD_max_ (Figure 5C). The SMA slope of this relationship did not differ significantly from 1 (b = 0.88, confidence intervals (CI): 0.6–1.2) and the intercept not significantly from 0 (a = 12.2, CI: −31.5–55.9). In Lake Washington the Lake Constance *Daphnia* phenology model explained 39% of the variation in TD_max_ (Figure 5D). However, in this case the MA slope significantly deviated from the 1∶1 line (slope: 1.8, CI: 1.3–2.4, intercept: −120, CI: −203–−37).

### Tests with Data Across the Northern Hemisphere

**Figure 6:**
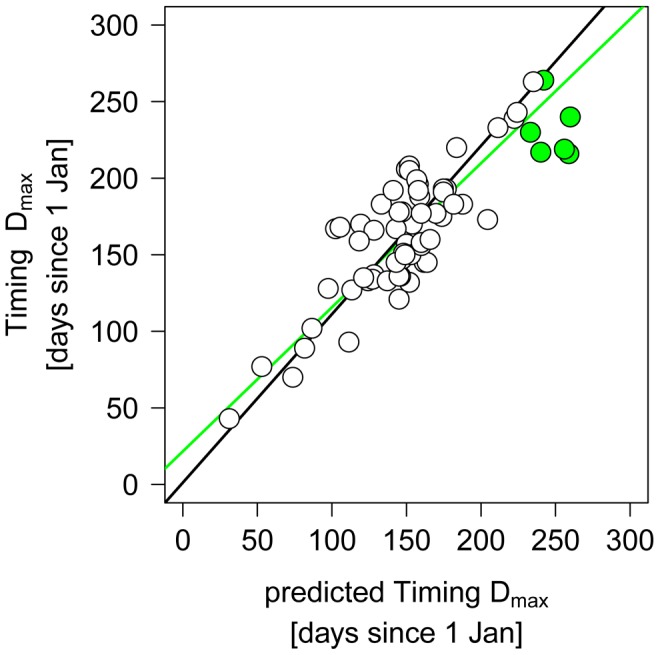
Relationship between observed and predicted timing of the *Daphnia* maximum in the NH data set. Green dots represent those lakes in which maximum temperatures did not reach 13°C. Lines represent the fits of SMA regression models. The black line shows the fit for only those lakes, respectively sites in which maximum temperatures reached 13°C (white dots), the green line presents the fit for all lakes in the data set, i.e., including the lakes (green dots) in which prediction is based on the timing of maximum water temperatures.

The LC *Daphnia* phenology model also performed well for predicting TD_max_ within the Northern Hemisphere data set (Figure 6). A linear regression between observed and predicted TD_max_ explained 57% of the variability observed among 66 *Daphnia* phenologies in 49 sites; that is, the model was successful also at latitudes and elevations differing strongly from Lake Constance. The slope of the SMA regression did not significantly differ from 1 (slope = 0.94, CI: 0.83–1.07), whereas the intercept was slightly positive (intercept = 21.6, CI: 1.9–41.3). When ignoring those lakes where maximum temperature did not surpass 13°C slope and intercept did not differ significantly from 1 (slope = 1.12, CI: 0.94–1.33), respectively 0 (intercept = −1.8, CI: −30.6–27.0). Residuals from the SMA regression did not show any relationship with latitude nor elevation (all p>0.05).

Furthermore, in linear models TT_13,0–5_ explained a slightly higher amount of variation in TD_max_ as latitude and elevation and clearly outcompeted this model based on AICc (Table 4, difference in AICc >8). However, when including elevation or elevation and latitude in addition to TT_13_ as independent factors, model performance was further enhanced, but the amount of explained variation only increased to 78% compared to 73% simply using TT_13,0–5_ as a predictor. Similar results were obtained when using a reduced data set in which those lakes were excluded which did not surpass a maximum temperature of 13°C (Table 4).

**Table 4:** Comparison of linear models relating the timing of the *Daphnia* maximum to the 13°C phenology (TT_13,0–5_), latitude (lat) and elevation (elev) of the respective lakes in the Northern Hemisphere for a) the complete data set (n = 66), and b) the data set including only those observations (seasons) in which maximum temperatures >13°C were observed (n = 62).

	a) complete data set		b) only observations with maximum temperatures >13°C	
Independent variables	Pseudo-R^2^	AICc	Pseudo-R^2^	AICc
TT_13, 0–5_, lat, elev,	0.78	601.6	0.75	546.5
TT_13, 0–5,_ elev	0.74	609.6	0.72	552.0
TT_13, 0–5_	0.73	609.8	0.70	552.9
TT_13, 0–5_, lat	0.73	610.9	0.71	554.4
lat, elev	0.70	618.1	0.64	565.6

All models were calculated with autocorrelated errors.

## Discussion

We developed a simple phenological model from a single, well-studied lake to predict the phenology of *Daphnia* maxima in lakes of the Northern Hemisphere. Our phenology model was established with data from Lake Constance, a large, deep and warm-monomictic lake during a period in which the lake’s trophic status changed from eutrophic to oligotrophic conditions. However, there was no evidence that the change in trophic status influenced the temperature-phenology relationship in Lake Constance as e.g., early and late TD_max_ were observed during eutrophic and more recent oligotrophic conditions. Likewise *Daphnia* spring phenologies followed vernal warming during most recent years in which rather extreme interannual variability in vernal water temperature increase was observed [25]. The robustness of the LC *Daphnia* phenology model against changes in trophic status was further supported by the tests of the relationship with long-term data from Lake Washington and Müggelsee; in both lakes the SMA residuals did not show any trend, despite both lakes experienced a reduction of nutrient inputs during the respective study periods, from eutrophic to mesotrophic conditions in Lake Washington [26] and from hypertrophic to eutrophic conditions in Müggelsee [27].

The applicability of our model to a large number of lakes supports the work of Gillooly & Dodson (2000) suggesting that temperature is indeed a major driver of *Daphnia* dynamics during spring and early summer [13]. However, the data from the three long-term studies as well as from the NH data set indicate that the mean temperature at TD_max_ is somewhat less than observed by Gillooly & Dodson (2000) in their smaller data set (n = 27, 18.5°C ±3.1 SD) – but within the standard deviation given in their study.

The high importance of temperature in regulating *Daphnia* dynamics during the spring period is probably due the fact that this period of exponential growth may be characterised as a period of low biological, i.e. bottom-up and top-down control. During the spring bloom, algal food is usually present in high concentrations, whereas predation is still low because most young-of-the year fish are not yet large enough to consume *Daphnia* and invertebrate predators are not yet present in high concentrations [16]. In contrast, water temperatures during spring are still low. As a consequence, temperature variability is likely the most important factor determining *Daphnia* population growth rate and consequently TD_max_.

The general importance of temperature in regulating *Daphnia* during spring does however not explain why the 13°C timing proved particularly useful in predicting the TD_max_. Physiological rates, e.g., egg development and growth rates, of *Daphnia* do neither show any step-wise changes when temperatures surpass 13°C nor are at their maximum at 13°C (e.g., [28]) suggesting that the physiology of *Daphnia* is unlikely the cause for the predictive power of the 13°C phenology. Hence, its relevance is likely due to ecological factors reducing predictive power when temperatures in the upper 5 m of the water column either have not yet reached or have surpassed 13°C. Before TT_13,0–5_ is reached, there is obviously a longer period to go for TD_max_, which reduces predictive power. Furthermore, *Daphnia* population size is still very low, that is, temperature induced variability in population growth rate does not have a strong influence on population development assuming exponential growth. A low influence of winter temperatures on TD_max_ is also supported when comparing the average winter temperatures in Lakes Constance, Washington and Müggelsee, which range between <4 to >7°C. Winter water temperatures within this range seem not to influence strongly *Daphnia* winter dynamics. Note, however, that daphnid abundances until late March are often close or at their detection limit in the three lakes resulting into large standard errors of mean weekly abundances. Nevertheless, there seems to be no evidence that winter *Daphnia* dynamics differ between the lakes with the lowest (Müggelsee) and the highest (Lake Washington) winter temperatures. Consequently, we should not expect a strong effect of winter conditions on spring dynamics. This is also in line with a modelling study showing that *Daphnia* overwintering biomass does not have a strong effect on TD_max_ [14].

The decline of predictability of TT_0–5_ for temperatures >13°C might be because the exponentially growing *Daphnia* population has already attained high concentrations at TT_13,0–5_ and is shortly before suppressing their food algae, resulting in the clear water phase [5], [29] and consequently food limitation. This might suggest that with TT’s >13°C, the relative importance of temperature in controlling *Daphnia* population growth is reduced relative to the influence of food limitation and possibly also predation. As a consequence, the predictive power of TT_0–5′_s >13°C for TD_max_ possibly declines. Clearly, this hypothesis regarding the importance of TT_13,0–5_ will be difficult to test with field data. Rather, we hope that our results will stimulate modelling analysis, which could test this hypothesis explicitly.

We subjected our linear model to tests with long-term data from two lakes and with data from a collection of lakes distributed widely across different latitudes, longitudes and elevations. Small, shallow, polymictic and highly eutrophic Müggelsee can be considered in many aspects as a limnological antithesis to deep, monomictic, oligotrophic Lake Constance. Despite these differences, the LC *Daphnia* phenology model could explain a significant amount of the variation in TD_max_ in Müggelsee. This supports a previous study showing that spring *Daphnia* dynamics in these two lakes were synchronized by the North Atlantic Oscillation during the period 1979–1994 [30].

**Figure 7:**
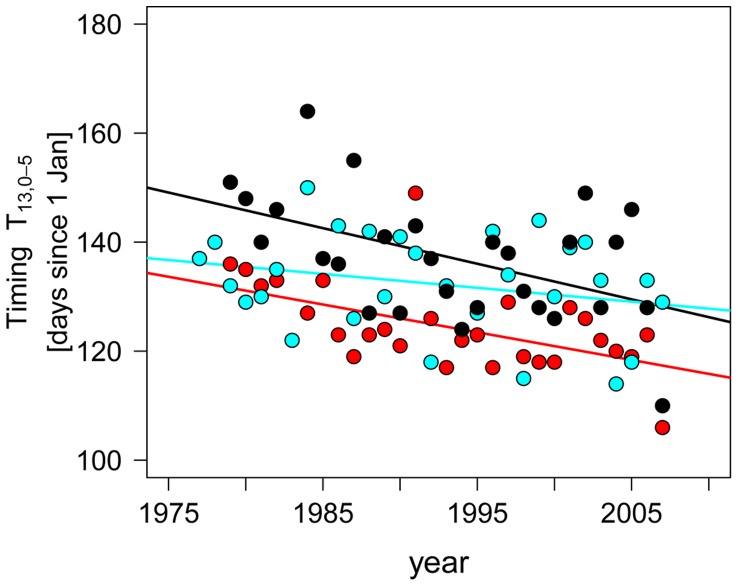
Changes in the timing of 13° phenology (TT_13_) in Lakes Constance (black dots, black line), Müggelsee (red dots, red line), and Washington (blue dots, blue line) during the last decades. Fits presents ordinary least-squares fits with slopes of −0.68 (0.22 SE), p<0.005 for Lake Constance, −0.54 (0.14 SE) p<0.001 for Müggelsee, and −0.1 (0.22 SE), ns for Lake Washington.

An even stronger test of the LC *Daphnia* phenology model seemed to be the Lake Washington data. In Lake Washington a climate induced mismatch between algae and *Daphnia* has developed with *Daphnia* unable to follow the temporal advancement of their algal food with earlier spring warming [9]. Furthermore, Winder and Schindler (2004a) stated that *“Daphnia* showed no response to water temperature variation (*P*>0.05)“ (but see [7]). In contrast, our study shows that the phenology of Lake Washington daphnids is strongly temperature controlled and can even be predicted by the LC *Daphnia* phenology model; i.e., a phenology model based on observed *Daphnia* dynamics from a lake several thousand km apart. Our study also provides a simple resolution to the observed paradox that *Daphnia* phenology in Lake Washington did not advance during recent decades despite a strong advance in Lake Washington timing of stratification and algal phenology [9] and the advance of *Daphnia* phenology in Lake Constance, Müggelsee and many other lakes [5], [11], [30]; TT_13,0–5_ did not significantly advance in Lake Washington (p>0.05) in contrast to lakes Constance and Müggelsee (Figure 7). That is, the difference in the response of *Daphnia* phenology to warming between Lake Washington and other lakes seems not be due to a different temperature dependency of *Daphnia* phenology caused by e.g., photoperiod dependency of resting egg hatching [9] or different temperature adaptation of *Daphnia* [7], but because there has not been a systematic trend towards warming in Lake Washington during the critical time period determining *Daphnia* phenology. This example shows that our approach based on temperature phenologies is an important tool to understand and predict phenology responses under current and future climate.

Although, the LC *Daphnia* phenology model proved to be highly successful in predicting temporal and spatial variation in TD_max_, TT_13, 0.5_ could only partially explain the variation in TD_max_. Residual variation might be due to other factors influencing *Daphnia* spring dynamics in addition to water temperatures (see below) or because of lack of information and detailed data: First, *Daphnia* – algae interactions are highly dynamic and it can be difficult to determine phenologies based on sampling programs with a weekly or especially fortnightly resolution [31]. Second, although there is a high spatio-temporal covariation of water temperatures during spring, not all variability will be covered by an analysis of one temperature phenology. For example, it is easy to imagine a situation where vernal water temperatures increase towards 12°C but not any further during the next e.g., two weeks because of cold weather. In such a case, the *Daphnia* population would still grow relatively fast towards its maximum, but our prediction of TD_max_ could be off by at least two weeks. Third, for the lakes covered in our literature study, no depth profiles are available and hence we included also studies in our NH data which did not report temperature dynamics in the 0–5 m layer, but at the surface or in the epilimnion (Table S1). Finally, we do not know the depth range occupied by *Daphnia* in specific lakes. For example, while we are sure that all daphnids in shallow Müggelsee are located in the upper 5 m of the water column, this is most likely not the case in the deep Lakes Washington and Constance. Consequently, in these lakes some unknown part of the *Daphnia* population will experience lower temperatures than those in the upper 5 m of the water column. Hence, one reason for residual variability could be a difference in depth range occupied by daphnids and consequently in the mean temperature experienced by the *Daphnia* population. The existence of these “methodological” inconsistencies makes the predictive power of the LC *Daphnia* phenology model even more remarkable.

Besides methodological inconsistencies, residual variability might clearly be caused by factors other than temperature influencing spring dynamics of *Daphnia*, e.g., predation, food quantity, quality and phenology and adaptation of *Daphnia* population to different temperature regimes. In this respect it is important to note that with respect to Lake Washington the SMA regression between predicted and observed TD_Max_ revealed a significant bias. Hence, it is a topic for future work to study whether this bias is due to e.g., the phenological mismatch of *Daphnia* with algae [9] or due to some other yet unknown factor. For example, a simulation approach has shown that *Daphnia* phenology is, besides water temperature, most strongly controlled by *Daphnia* mortality rates, i.e., predatory losses [14]. In addition, in very oligotrophic lakes *Daphnia* phenology may be shifted towards summer because of strong food limitation [10]. These factors influencing *Daphnia* phenology may be better identified and quantified after accounting for the effect of temperature with the *Daphnia* phenology model established in this study.

In addition to food web interactions, temperature adaptation of daphnids among and within species might be expected to cause systematic deviation of observed TD_max_ versus predicted TD_max_ in the NH data set. For example, a possible adaptation of daphnids in lakes at high latitudes to lower temperatures should result in an earlier TD_max_ compared to the prediction based on the data from temperate Lake Constance. However, the MA slope between observed and predicted TD_max_ is very close to one, and residuals from this relationship do not show a significant relationship to latitude. This suggests that temperature adaptation of daphnids seems not to strongly influence *Daphnia* phenology. This suggestion is in line with the failure to detect thermal adaptation experimentally with pond *Daphnia magna* clones sampled across a latitudinal gradient from Spain to Finland [32]. In these intermittent populations of *D. magna* the lack of thermal adaptations was suggested to be due to obligate diapause precluding the necessity for thermal adaptation, as adverse temperature conditions can be avoided by entering diapause. However, it could also be argued that adverse temperatures in many larger water bodies with permanent *Daphnia* populations can be avoided too by daphnids as those larger water bodies usually do not warm as strongly as small ponds and often offer vertical gradients of water temperature, which may be used by daphnids if surface temperature increase beyond optimal levels. Overall, our results suggest that thermal adaptation of *Daphnia* populations (if any) is not strong enough to substantially influence *Daphnia* phenology in permanent water bodies. However, clearly more data on *Daphnia* phenology are needed in especially boreal and mediterranean climatic regions to provide a stronger test of a potential influence of *Daphnia* temperature adaptation on the phenology of *Daphnia* populations.

Our model relies on temperature phenology as a predictor variable and not on the temperature average during a distinct period prior to the phenological event, which is the predictor usually used to relate phenological change to warming [7], [33]–[35]. For temperate lakes, for example, May temperatures do also have a high predictive power for TD_max_. However, this commonly used temperature average approach [7], [16] may be less able to predict phenologies beyond the temperate zone and beyond the current climate, as the temperatures in a specific seasonal time period, e.g. average May temperature, might not be relevant for a specific phenology in more southern latitudes or in a future warmer climate, when e.g. variability in April temperatures might be more important. Likewise it will not be possible to predict interannual variability in TD_max_ in high elevation or latitude lakes with average May temperatures, when the lake is still frozen in May and temperature variability is very low. In contrast, our results show that an approach based on temperature phenologies can be applied also to latitudes and elevations beyond a specific climatic zone, and hence may have a large potential also for other phenological studies.

To conclude, we show that the vernal increase in temperature is indeed an important factor affecting *Daphnia* growth rates during spring in lakes of the Northern Hemisphere and that it is possible to predict *Daphnia* phenology across these lakes with a temperature relationship based on long-term phenology data of a single lake. Other potentially important factors such as variability in food quantity, quality and/or predation pressure or temperature adaptation of *Daphnia* seem to play only a secondary role. This suggests that *Daphnia* phenology will respond strongly and immediately to climate warming provided that there is warming during the seasonal time period critical for *Daphnia* phenology. The established phenology model in combination with hydrodynamical modelling of water temperatures should be a valuable tool to predict the magnitude of the response of *Daphnia* phenology in lakes of the Northern Hemispheric under future warming scenarios.

## Supporting Information

Table S1 **Information on lakes in the Northern Hemisphere data set.**
(PDF)
