# Supplementary material for: Uniform Temperature Dependency in the Phenology of a Keystone Herbivore in Lakes of the Northern Hemisphere
Source: PLoS One. 2012 Oct 5;7(10):e45497. doi: 10.1371/journal.pone.0045497 (PMC3465311; doi:10.1371/journal.pone.0045497)
Supplement: Table S1 — Information on lakes in the Northern Hemisphere data set. (PDF) [file pone.0045497.s001.pdf]

Uniform temperature dependency in the phenology of a keystone herbivore in lakes of the Northern Hemisphere

Dietmar Straile, Rita Adrian & Daniel E. Schindler

Table S1 Information on lakes in the Northern Hemisphere data set

This table provides latitudes, altitudes and water layers depths of temperatures reported in the individual publications, and sources of the data. Note that the water layer depth reported varies strongly between studies and that this will contribute to the unexplained variability of the phenology model.

Lake	latitude [°N]	elevation [m]	water layer of reported temperatures	Source
Ardleigh Reservoir	5	20	0 - 4 m	Abdul-Hussein & Mason 1987
Artificial Pond	36.02	25	0 - 1.4 m	Shei et al. 1988
Aziscoos Lake	45	460	surface	Tappa 1965
Bantam Lake	41.42	272	surface	Brooks 1946
Base Line Lake	42.26	259	surface	Hall 1964
Eglwys Nynydd	51.32	10	surface	George & Edwards 1974
Fort Whyte Lakes (II,III,IV)	49.49	230	surface	Depuis & Hann 2009
Großer Vätersee	53	60	2 m	Steiner 2002
Greifensee	47.33	435	0 - 5m	Mittelholzer 1970
Gronne	48.44	478	surface	Maier 1996
Groß-Glienicker See	52.28	31	surface	Deneke 2001
Hangdog Pond	45.37	190	0 - 1.7 m	Kwik & Carter 1975
Keeple Lake	47.26	700	0.5 m	Hazelwood & Parker 1961
Klostersee	47.58	533	surface	Seitz 1980
Lago Paione Superiore	46.1	2269	1 m	Callieri et al, 2006
Lago Trasimeno	43.08	259	0-5	Taticchi et al. 1991
Lago di Arancio	37.38	180	surface	Naselli-Flores & Barone 1997
Lake Buttermere	54.32	100	3 m	Smyly 1979
Lake Czarny	49.11	1583	0-5	Gliwicz et al. 2001
Lake Erken	59.5	11	0-epilimnion	Nauwerck 1963
Lake Esrom	56	17	surface	Petersen 1983
Lake Imikpuk	71.2	1	surface	Edmondson 1955
Lake Kvernavatnet	60.6	37	surface	Borsheim et al. 1988
Lake Lacawac	41.23	438	surface	Tessier 1986
Lake Lombola	69.07	214	0-epilimnion	Primicerio & Klemetsen, 1999
Lake Mekkojärvi	61.13	130	surface	Ojala & Salonen 2001
Lake Mitchell	43.47	449	0 - 3 m	DeMott 1983
Lake Morskie Oko	49.11	1395	0 - 5 m	Gliwicz et al. 2001
Lake Myvatn	63.35	278	1 m	Adalsteinsson 1979
Lake Oglethorpe	33.52	182	surface	Saunders et al. 1999
Lake Reading	38.24	350	0 - 2 m	Brunner 1984
Lake Sobygard	56.25	49	0 - 1 m	Jeppesen et al. 1990
Lake Thingvallatan	64.1	101	0-2m	Antonsson 1992
Lake Vechten	52.04	4	surface	Hovenkamp 1990

Lake Vela	40.16	45	0 - 1 m	Castro & Goncalves 2007
Lake Vesijärvi	61.01	81	surface	Nykänen et al. 2009
Lake Volkerak	51.38	-1	0 - 5 m	Mooji et al. 2003
Oberer Arosasee	46	1800	surface	Winder et al. 2003
Oneida Lake	43.2	34	2 m	Mills et al. 1978
Professoren Weiher	48.44	478	surface	Maier 1996
Richter Lake	45.12	438	0 m	Gannon et al 1984
Rio Seco Lake	37	3040	0 - 2.9 m	Perez-Martinez et al. 2007
Saidenbach Reservoir	50.44	439	mixed layer	Horn & Benndorf 1980
Saidenbach Reservoir	50.44	439	0 - 5 m	Pinkhaus et al. 2007
Stockton Lake	37.41	264	0 - 5 m	Havel & Graham 2006
Tjeukemeer	52.5	-1	0 -1.5 m	Vijverberg 1974
Waldsea Lake	52.17	550	surface	Swift & Hammer 1979
Weidsee	47.58	533	0 - 7 m	Jacobs 1977

References

Adalsteinsson H. (1979) Zooplankton and Its Relation to Available Food in Lake Myvatn. *Oikos* 32, 162-194

Antonsson U. (1992) The Structure and Function of Zooplankton in Thingvallavatn, Iceland. *Oikos* 64, 188-221

Borsheim K.Y., Andersen S., Johnsen G.H., Kambestad E.O. & Norland S. (1988) Primary and bacterial production compared to growth and food requirements of Daphnia longispina in Lake Kvernavatnet, west Norway. *J.Plankt.Res.* 10, 921-939

Bruner G.J. (1984) Coexistence strategies of Daphnia in Lake Reading Ks. *Emporia Sate Research Studies* 32, 5-21

Callieri C., Corno G. & Bertoni R. (2006) Bacterial grazing by mixotrophic flagellates and *Daphnia longispina*: a comparison in a fishless alpine lake. *Aquatic Microbial Ecology* 42, 127-137

Castro B.B. & Goncalves F. (2007) Seasonal dynamics of the crustacean zooplankton of a shallow eutrophic lake from the Mediterranean region. *Fundamental and Applied Limnology* 169, 189-202

DeMott W.R. (1983) Seasonal succession in a natural *Daphnia* assemblage. *Ecol.Mon.* 53, 321-340

Deneke R. (2002) Untersuchungen zum Einfluß des Schichtungsverhaltens (Mixis) auf die Trophie und Planktonsukzession in eutrophen Seen unter besonderer Berücksichtigung des Klarwasserstadiums im Frühjahr. PhD thesis, Brandenburgische Technische Universität Cottbus.

Dupuis A.P. & Hann B.J. (2009) Warm spring and summer water temperatures in small eutrophic lakes of the Canadian prairies: potential implications for phytoplankton and zooplankton. *Journal of Plankton Research* 31, 489-502

Edmondson W.T. (1955) The Seasonal Life History of *Daphnia* in An Arctic Lake. *Ecology* 36, 439-455

Gannon J.E., Cap R.K., Stewart K.M. & Stewart W.A. (1984) Zooplankton community composition in Richter Lake, Wisconsin. *Verh.Internat.Verein.Limnol.* 22, 525-532

George D.G. & Edwards R.W. (1974) Population dynamics and Production of *Daphnia hyalina* in A Eutrophic Reservoir. *Freshw.Biol.* 4, 445-465

Gliwicz Z.M., Slusarczyk A. & Slusarczyk M. (2001) Life history synchronization in a long-lifespan single-cohort *Daphnia* population in a fishless alpine lake. *Oecologia* 128, 368-378

Hall D.J. (1964) Experimental Approach to Dynamics of Natural-Population of *Daphnia galeata mendotae*. *Ecology* 45, 94-112

Havel J.E. & Graham J.L. (2006) Complementary population dynamics of exotic and native Daphnia in North American reservoir communities. *Archiv fur Hydrobiologie* 167, 245-264

Hazelwood D. & Parker R.A. (1961) Population-Dynamics of Some Fresh-Water Zooplankton. *Ecology* 42, 266-274

Horn W. & Benndorf J. (1980) Field investigations and model simulation of the dynamics of zooplankton populations in fresh waters. *Int.Rev.ges.Hydrobiol.* 65, 209-222

Hovenkamp W. (1990) Population dynamics of coexisting *Daphnia* species in Lake Vechten. 1-94. PhD thesis, University of Amsterdam.

Jacobs J. (1977) Coexistence of similar zooplankton species by differential adaptation to reproduction and escape in an environment with fluctuating food and enemy densities. *Oecologia* 30, 313-329

Jeppesen E., Sondergaard M., Sortkjaer O., Mortensen E. & Kristensen P. (1990) Interactions Between Phytoplankton, Zooplankton and Fish in A Shallow, Hypertrophic Lake - A Study of Phytoplankton Collapses in Lake Sobygard, Denmark. *Hydrobiologia* 191, 149-164

Kwik J.K. & Carter J.C.H. (1975) Population-Dynamics of Limnetic Cladocera in A Beaver Pond. *Journal of the Fisheries Research Board of Canada* 32, 341-346

Maier G. (1996) Daphnia invasion: Population dynamics of Daphnia assemblages in two eutrophic lakes with particular reference to the introduced alien Daphnia ambigua. *Journal of Plankton Research* 18, 2001-2015

Mason C.F. & Abdulhussein M.M. (1991) Population dynamics and production of *Daphnia hyalina* and *Bosmina longirostris* in a shallow, eutrophic reservoir. *Freshwater Biology* 25, 243-260

Mills E.L., Forney J.L., Clady M.D. & Schaffner W.R. (1978) Oneida Lake. In: *Lakes of New York state, Vol. 2: Ecology of the lakes of Western New York* (ed. Bloomfield J.A.), pp. 367-451. Academic Press, New York.

Mittelholzer E. (1970) Populationsdynamik und produktion des Zooplanktons im Greifensee und im Vierwaldstättersee. *Schweizerische Zeitschrift für Hydrologie* 32, 91-148

Mooij W.M., Hülsmann S., Vijverberg J., Veen A. & Lammens E.H.R.R. (2003) Modeling *Daphnia* population dynamics and demography under natural conditions. *Hydrobiologia* 491, 19-34

Naselli-Flores L. & Barone R. (1997) Importance of water-level fluctuation on population dynamics of cladocerans in a hypertrophic reservoir (Lake Arancio, south-west Sicily, Italy). *Hydrobiologia* 360, 223-232

Nykanen M., Vakkilainen K., Liukkonen M. & Kairesalo T. (2009) Cladoceran remains in lake sediments: a comparison between plankton counts and sediment records. *Journal of Paleolimnology* 42, 551-570

Ojala A. & Salonen K. (2001) Productivity of *Daphnia longispina* in a highly humic boreal lake. *Journal of Plankton Research* 23, 1207-1215

Perez-Martinez C., Barea-Arco J., Conde-Porcuna J.M. & Morales-Baquero R. (2007) Reproduction strategies of Daphnia pulicaria population in a high mountain lake of Southern Spain. *Hydrobiologia* 594, 75-82

Petersen F. (1983) Population dynamics and production of *Daphnia galeata* (Crustacea, Cladocera) in Lake Esrom. *Hol.Ecol.* 6, 285-294

Pinkhaus O., Schwerin S., Pirow R., Zeis B., Buchen I., Gigengack U., Koch M., Horn W. & Paul R.J. (2007) Temporal environmental change, clonal physiology and the genetic structure of a *Daphnia* assemblage (*D. galeata-hyalina* hybrid species complex). *Freshw.Biol.* 52, 1537-1554

Primicerio R. & Klemetsen A. (1999) Zooplankton seasonal dynamics in the neighbouring lakes Takvatn and Lombola (Northern Norway). *Hydrobiologia* 411, 19-29

Saunders P.A., Porter K.G. & Taylor B.E. (1999) Population dynamics of Daphnia spp. and implications for trophic interctions in a small, monomictic lake. *J.Plankt.Res.* 21, 1823-1845

Seitz A. (1980) The coexistence of three species of *Daphnia* in the Klostersee 1. Field studies of three species of *Daphnia* in the Klostersee. *Oecologia* 45, 117-130

Shei P., Iwakuma T. & Fujji K. (1988) Population dynamics of *Daphnia rosea* in a small eutrophic pond. *Ecol.Res.* 3, 291-304

Smyly W.J.P. (1979) Population dynamics of *Daphnia hyalina* Leydig (Crustacea, Cladocera) in A Productive and An Unproductive Lake in the English Lake District. *Hydrobiologia* 64, 269-278

Steiner S. (2002) Dynamik und Energietransfer einer planktischen Crustaceengemeinschaft in Abhängigkeit von der Nahrungsgrundlage und den Planktivoren. 1-145. PhD thesis, Technical University Dresden.

Swift M.C. & Hammer U.T. (1979) Zooplankton Population-Dynamics and Diaptomus Production in Waldsea Lake, A Saline Meromictic Lake in Saskatchewan. *Journal of the Fisheries Research Board of Canada* 36, 1431-1438

Tappa D.W. (1965) The dynamics of the association of six limetic species of *Daphnia* in Aziscoos lake. Maine. *Ecol.Mon.* 35, 395-423

Taticchi M.I., Artegiani I. & Isa C. (1991) Cladocera populations in Trasimeno Lake (Italy): 17/07/87 to 28/12/88. *Verh.Internat.Verein.Limnol.* 24, 1049-1057

Tessier A.J. (1986) Comparative population regulation of two planktonic cladocera (*Holopedium gibberum* and *Daphnia catawba*). *Ecology* 67, 285-302

Vijverberg J. & Richter A.F. (1982) Population-Dynamics and Production of Daphnia-Hyalina Leydig and Daphnia-Cucullata Sars in Tjeukemeer. *Hydrobiologia* 95, 235-259

Winder M., Bürgi H.R. & Spaak P. (2003) Mechanisms regulating zooplankton populations in a high-mountain lake. *Freshwater Biology* 48, 795-809
